# Averaging of Backscatter Intensities in Compounds

**DOI:** 10.6028/jres.107.046

**Published:** 2002-12-01

**Authors:** John J. Donovan, Nicholas E. Pingitore, Andrew J. Westphal

**Affiliations:** Department of Geological Sciences, The University of Oregon, Eugene, OR 97403-1272; Department of Geological Sciences, The University of Texas at El Paso, El Paso, TX 79968-0555; Department of Physics, The University of California, Berkeley, CA 94720-4767

**Keywords:** atomic fraction, atomic number correction, backscatter, elastic scattering, electron fraction, electron scattering, mass averaging, mass effect, mass fraction, microanalysis, multi-element compounds, quantitative microanalysis

## Abstract

Low uncertainty measurements on pure element stable isotope pairs demonstrate that mass has no influence on the backscattering of electrons at typical electron microprobe energies. The traditional prediction of average backscatter intensities in compounds using elemental mass fractions is improperly grounded in mass and thus has no physical basis. We propose an alternative model to mass fraction averaging, based of the number of electrons or protons, termed “electron fraction,” which predicts backscatter yield better than mass fraction averaging.

## 1. Introduction

Calculations of average backscatter (or electron loss) for compounds in electron probe microanalysis (EPMA) have traditionally utilized mass fraction averaging [1]. For multi-element samples the calculations of average mass absorption coefficients and average stopping power are properly formulated using mass fractions in traditional expressions because the terms are grounded in mass units. The same cannot be said of the average backscattering loss factor, *R*, which is generally assumed to be mass-dependent by Castaing [2], Heinrich [3], Duncumb and Reed [4], and Joy [5] for inter-element effects by the use of the expression,

$$R_{i}=\sum_{j}c_{j}R_{ij}$$ (1)

where *c_j_* is the mass fraction and *R_ij_* is the backscatter loss factor for element *i* in the presence of element *j* in a multi-element sample. Although there have been attempts in the literature to find alternative methods based on various formulation involving atomic fractions these have systematically yielded even worse results. Reports of difficulty [6] with some Si-Pb and other compounds where a large atomic number correction is necessary suggest the need to re-examine these assumptions.

### 1.1 Physics of Electron Backscatter

Electron backscatter is primarily the result of the electrostatic interaction of incident electrons with the Coulombic field of the atom (essentially the positive charge of the nucleus), which in turn is produced by the total charge of the protons (partially modified by the screening effect of the inner orbital electrons), which is related to the number of each, that is *Z*. The electromagnetic dipole component is unlikely to provide more than a negligible contribution to backscatter, especially in non-magnetic materials where this property is effectively randomized. Therefore some variety of *Z*-based averaging should, in principle, apply for calculations involving multi-element compounds. That is to say, neutrons, which have no electric charge, only mass, should have no effect on productions of this type at typical electron probe microanalysis (EPMA) energies and precision levels. But mass fraction averaging is based on atomic weight, which is the total mass of the protons, electrons and neutrons.

Furthermore, from a physical perspective, it is unlikely for incident electrons at energies typically attained in EPMA to measurably interact with the neutron of an atom. In fact the wavelength of a 100 keV electron is some 10^4^ times larger than the interaction volume of the neutron. Even more to the point, it is uncontroversially accepted that electromagnetic effects will dominate over gravitational effects (the only known intrinsic property of mass besides nuclear spin) in this atomic regime by a factor of approximately 10^40^.

### 1.2 Scope of This Study

In an effort to detect any possible effect due solely to atomic weight, as opposed to atomic number, we performed low uncertainty measurements of absorbed current in samples in which the *only* difference was mass, that is the number of neutrons. Specifically, we examined stable isotopes of the same element. For this experiment, we compared samples of normal Cu (mass 63.54), and enriched 65Cu; normal Ni (mass 58.71) and enriched ^60^Ni; and normal Mo (mass 95.94) and enriched ^100^Mo.

If mass, represented by the presence of the neutron, affects the production of backscatter, then we would expect to see a measurable difference in the absorbed currents between these stable isotope pairs. Absorbed current is, of course, related to backscatter by the simple relation,

$$\eta =\frac{i_{\text{absorbed}}}{i_{\text{beam}}}$$ (2)

where *i*_beam_ is the measured beam current and *i*_absorbed_ is the measured absorbed or specimen current.

Low uncertainty absorbed current measurements were also performed on the NIST Au-Cu-Ag alloys to evaluate a number of expressions in predicting average backscatter yield by interpolating from pure element end-members.

## 2. Experimental

### 2.1 Electron Microprobe Conditions

All measurements were made on a Cameca^1^ SX-51 electron microprobe at the University of California at Berkeley, Department of Earth and Planetary Science. The conditions for the absorbed and beam current measurements were 15 keV, 100 nA. A total of 15 measurements were averaged for each data point plotted and each measurement is itself the average of 5 A/D current conversions. Where error bars are not shown in the data figures, one standard deviation is smaller than the symbol size.

### 2.2 Backscatter Measurements

Care was taken to reduce or correct for both the additional contribution of absorbed current from reabsorbed secondary electrons produced by backscattered electrons striking the sample chamber walls and the loss of secondary electrons from the target area. This was accomplished by the use of a small bias of 22.5 V applied to a separately insulated area surrounding the sample [3].

Sample voltage biasing is usually necessary for accurate determination of absolute backscatter coefficients. However, to compare the relative merit of various average atomic number models, we found the precision of the measurement to be more critical. Since the contribution of secondary electrons is very small for electrically insolated targets of minimal size (<10 mm3) and also fairly constant over large ranges of atomic number, we established that sample biasing was unnecessary in comparing stable isotopes pairs where the atomic numbers (and hence the nuclear charges) are *exactly* the same. In this paper, results reported in absorbed current were generally not acquired using a voltage biased sample mount, while those results reported in backscatter coefficient, (*η*), were acquired using a voltage biased sample mount.

## 3. Results

**Fig. 1:**
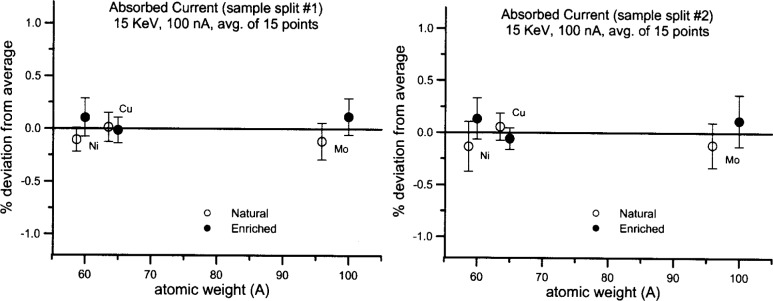
Absorbed current intensities (analog to backscatter yield) acquired on three stable isotope/natural abundance pairs. The fractional atomic weight numbers are averages for natural abundance isotopic mixtures, presented for comparison with masses for enriched isotopes. Each point represents an average of 15 measurements, shown relative to the average intensity measured for both natural and enriched isotopes; each error bar is one standard deviation. The complete analysis (Measurement #1) was repeated for verification on a second probe mount of a separate set of isotope pairs, and this second set of results is presented as Measurement #2. Note that all the measurements fall within 0.25 % of the respective average of each isotope pair, and that even the one-standard-deviation error bars are within 0.5 % of the average. This result indicates that any possible mass effect on the production of backscatter electrons is significantly less than the difference in mass between the isotope pairs.

Figure 1 presents high-precision results for absorbed current, measured on two different sample splits of the isotope pairs. The variation (~0.2 %) within the pairs is similar to the precision level, that is, roughly an order of magnitude smaller than the differences in mass between the isotope pairs. The differences in atomic mass between the natural abundance and enriched isotopes range from 2.2 % to 4 %.

If mass did affect pure element backscatter intensities, one might have expected an increase in backscatter of about 2.2 % per atomic mass unit (u) in the region of Ni and Cu (based on pure Fe and Cu measurements) and about 0.22 % per atomic mass unit (u) in the region of Mo (based on Cu and Ag measurements). Given the respective differences in the Ni, Cu and Mo isotope pairs of 1.29 u, 1.46 u, and 4.06 u, we might have expected to observe backscatter intensity differences on the order of 2.8 %, 3.2 % and 0.9 % for Ni, Cu and Mo, respectively.

The observed differences in the isotope pairs were approximately 5 to 15 times *smaller* than these mass-effect calculations suggest. Furthermore the minuscule variation of backscatter with mass appears random, and likely represents experimental error. We must conclude that mass, represented here by the additional atomic mass of neutrons, does not affect backscattering of electrons under microprobe conditions. Mass therefore should not appear as a term in EPMA models that predict average backscatter.

## 4. Discussion

### 4.1 Averaging From Pure Elements to Predict Properties of Compounds

It is well known that atomic fraction averaging (the ratio of the number of atoms in a compound) poorly predicts the properties of compounds under electron bombardment. For example, uranium sulfide, US, exhibits properties more similar to those of uranium that those of sulfur, even though the atomic proportion of the two elements is 1:1. Mass-averaging of element properties became established early in the history of electron probe microanalysis because of its reasonable success in predicting the properties of compounds from the observed properties of the relevant pure elements.

### 4.2 Electron Fraction Averaging

Physical considerations and the isotope data presented above suggest the use of electron fraction based averaging [7, 8, 9, 10]. The electron fraction is the fraction of the electrons, or protons, in a compound contributed by each of the elements present. The electron fraction is calculated as:

zi=aiZi∑i=1naiZi (3)

where, *a_i_* is the atomic fraction and *Z_i_* is the atomic number of element *i* in the compound. The difference between this expression and mass fraction is the substitution of atomic number for atomic weight.

The variation in *A/Z* in natural elements is as much as 30 % (over several hundred percent for hydrogen and helium). Some elements have more neutrons (and hence more mass) than might be expected from their atomic number, while others have fewer neutrons (and hence less mass) than expected.

Mass fraction averaging in traditional models thereby imposes a systematic error on backscatter averaging, an error that is described by the variation of *A/Z* vs *Z* for the natural elements. This mass-induced (or neutron induced) error depends on the specific ratios of *A/Z* for the elements of the compound in question. The difference between the mass fraction and electron fraction for many compounds is 1 % to 3 %, but it can exceed 20 % to 25 %, e.g., lead sulfide or uranium carbide (Table 1).

### 4.3 Methods to Compare Mass and Electron Fraction Averaging for Backscatter Prediction

There are two distinct approaches to comparing the relative merit of the two fractional models. One is to predict the property of the compound from the weighted (by mass, electron, or whatever) average of the properties of the relevant pure elements, and compare this to the value of the property measured on the compound. This *property averaging* method has been widely used in estimations of average backscatter, based on mass averaging, by many early experimenters, although it was usually limited to mixtures of two elements.

The other method is to plot a series of measurements of the property versus calculated hypothetical average atomic numbers and observe the smoothness of fit to a simple polynomial or exponential curve. We term this *atomic number averaging.*

We will restrict the present discussion to the use of property averaging to evaluate the predictive powers of mass fraction and electron fraction averaging and deal with atomic number averaging in a separate paper.

### 4.4 Backscatter Prediction From Property Averaging

Predictions of the backscatter from intermediate compositions of Au-Cu-Ag alloys made using property averaged measurements from pure elements are performed using the expression for mass fraction [11, 4]:

$$\bar{\eta }c_{\text{AB}}=c_{A}\eta_{A}+c_{B}\eta_{B}$$ (4)

where *c*_A_ and *c*_B_ are the mass fractions of elements *A* and *B* in the binary compound and *η*_A_ and *η*_B_ are the backscatter ratios of the pure elements. The electron fraction property averaging expression for intermediate compositions derived from measurements on pure elements is similarly assumed to be:

$$\bar{\eta }z_{\text{AB}}=z_{A}\eta_{A}+z_{B}\eta_{B}$$ (5)

where *z*_A_ and *z*_B_ are the electron fractions of elements *A* and *B* in the binary compound from Eq. (3). In all cases, it is assumed that the mixing of binary end-member properties is on a straight line.

**Fig. 2:**
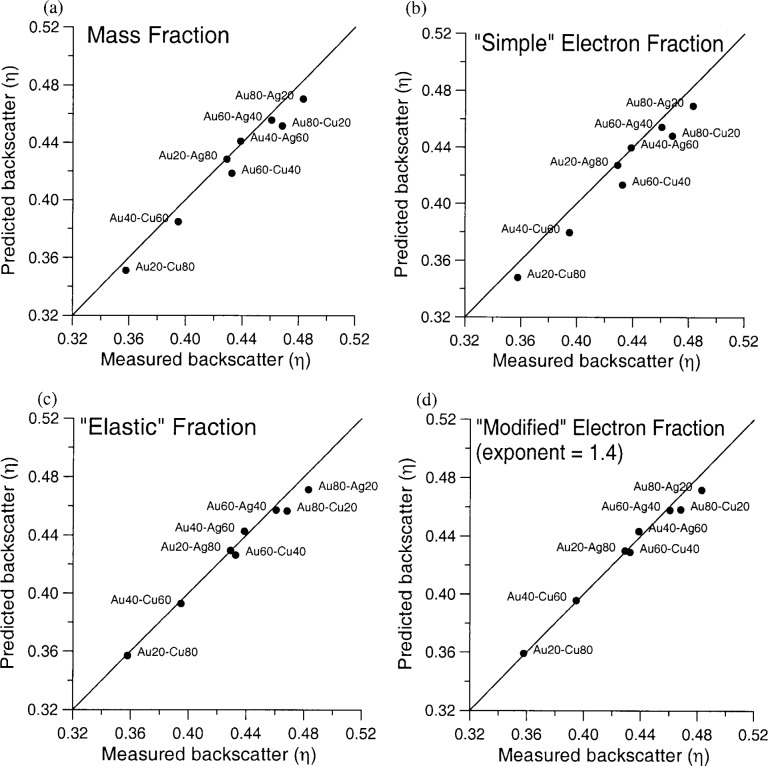
Property average predictions from pure element backscatter intensities versus backscatter measurements on NIST SRM 481/482 Au/Ag/Cu binary alloys (20 keV, 100 nA, average of 10 measurements per point, 22.5 V sample bias) for (a) mass fraction, Eq. (4), (b) simple electron fraction, Eq. (5), (c) elastic fraction, Eq. (9) and (d) “modified” electron fraction, Eq. (11). Both the elastic fraction and modified electron fraction predictions give good results to the data. The parameterized elastic fraction is mathematically equivalent to a *Z*^1.35^ function and therefore similar to a modified electron fraction using a *Z* exponent of 1.4, as is seen from the similarity of the two plots.

In Figs. 2a and 2b, mass and electron fraction property predictions give similar results, with a slightly better prediction from the mass fraction average.

### 4.5 Backscatter Prediction Based on Elastic Cross Section Averaging

Backscatter is an elastic scattering process, to a first order dependent on the number of protons in the nucleus and to a second order on its effective nuclear charge. At typical energies utilized in EPMA there is no interaction with neutrons, as demonstrated by the isotope data previously shown. The word effective denotes that the total nuclear charge is not involved in elastic scattering of incident electrons, especially for atoms of higher atomic number due to screening of the nucleus by the inner orbital electrons. Because of this nuclear screening effect, the effective charge of the nucleus is reduced and a correction is required to account for this.

The use of mass fraction for average backscatter calculations contains a fortuitous bias for nuclear screening due to the non-linearity of atomic weight with respect to *Z*. (*A* increases faster than *Z*, especially at high *Z*). This atomic weight scaling effect is produced by the additional mass of the neutron, and is completely unrelated to elastic scattering of electrons at EPMA energies.

Armstrong [12] noted that the ratios of elastic scattering cross section and atomic mass to atomic number correlate fairly well. Since the elastic scattering term is essentially the size of the target atom as seen by an electron beam (for backscattered electrons), Armstrong felt this might explain the observed correlation of various electron-solid interactions with mass fraction. Thus, the correlation of mass fraction with electron backscatter yield, demonstrated by Heinrich [11] and Colby [13], may be accidental. In fact, during efforts to create more physically based electron interaction models, this relative elastic scattering ratio has been suggested by others as one possible basis for calculating the elemental proportioning of electron backscatter in multi-element compounds, rather than the traditionally utilized mass fraction basis from Castaing and Heinrich.

Armstrong used the following expression for single elastic scattering that produces results that vary only slightly with the energy of the incident beam:

$$\sigma^{E}=5.21\times 10^{-21}\frac{Z^{2}}{E^{2}}\frac{4\pi }{\alpha \left(1+\alpha \right)}{\left(\frac{E+m_{0}c^{2}}{E+2m_{0}c^{2}}\right)}^{2}$$ (6)

where *E* is the electron energy in keV, *Z* is the atomic number, *m*_0_*c*^2^ ≈ 511 keV, and *α* is an effective nuclear charge screening factor,

$$\alpha =3.4\times 10^{-3}\frac{Z^{0.67}}{E}$$ (7)

from Newbury et al. [14]. To calculate an elastic scattering cross section fraction, we assume that the averaging is based on the additivity of the elastic scattering weighted atom proportion of each element in the compound. The elastic scattering fraction, is therefore,

$$\sigma_{i}=\frac{a_{i}\sigma_{i}^{E}}{\sum_{i=1}^{n}a_{i}\sigma_{i}^{E}}$$ (8)

where *a_i_* is the atomic proportion of the element in the compound, $\sigma_{i}^{E}$ is the total elastic scattering cross section for element *i* as defined in Eq. (6).

We calculate the elastic scattering cross section average, derived from Armstrong, as:

$$\bar{\eta }\sigma_{\text{AB}}=\sigma_{A}\eta_{A}+\sigma_{B}\eta_{B}$$ (9)

Where *σ*_A_ and *σ*_B_ are the elastic fractions of elements *A* and *B* in the binary compound from Eq. (8). It is assumed that the mixing of properties is on a straight line between pure element end-members.

In Figures 2a, 2b, and 2c, the best prediction is given by the elastic scattering fraction average, based on Eq. (9), derived from Armstrong.

### 4.6 Modified Electron Fraction Averaging

The simple (*Z^x^*, where *x* = 1.0) electron fraction model does not predict property averaged backscatter production in compounds quite as well as the elastic scattering fraction model. Nuclear screening by the inner orbital electrons, especially in nuclei of the higher *Z* elements, limits the performance of simple electron fraction averaging. The simple electron fraction model assumes that all protons (whose Coulombic field is the contributing factor for elastic scattering) are of equal influence. But as the inner orbital electrons screen the nucleus with increasing efficiency, the rate of increase in backscatter yield decreases significantly for the higher *Z* elements. Since the elastic scattering fraction formulation includes a correction for this, it predicts backscatter better. The mass fraction includes a bias in the proper direction due to the increase in neutron count in higher atomic number elements and so partially compensates for the screening effect, as noted by Armstrong.

With this screening effect in mind, we adjust the electron fraction calculation to compensate for a variation in scattering with *Z*. The calculation of this modified electron fraction is,

$$z_{i}^{\left(x\right)}=\frac{a_{i}Z_{i}^{x}}{\sum_{i=1}^{n}a_{i}Z_{i}^{x}}$$ (10)

where *x* is an exponent generally close to 1.0. The exponent (*x*) in parentheses simply indicates the derivation of the modified term. To utilize the modified electron fraction adjusted for nuclear screening effects in the calculation of property averaging, we use the following expression,

$$\bar{\eta }z_{\text{AB}}^{\left(x\right)}=z_{A}^{\left(x\right)}\eta_{A}+z_{B}^{\left(x\right)}\eta_{B}$$ (11)

where $z_{A}^{\left(x\right)}$ and $z_{A}^{\left(x\right)}$ are the modified electron fractions of elements *A* and *B* in the binary compound from Eq. (10).

Figure (2d) reveals that a good fit can be obtained with this simple adjustment where the best fit is obtained with an electron fraction exponent of *Z^x^*, where *x* = 1.4 for the NIST SRM 481/482 Au-Ag-Cu alloys and pure elements.

Although some deviation for the high Au compositions in the predicted backscatter data may be noted due to slight surface contamination of the pure Au standard by Cu and Ag during polishing (~1 % Cu as bulk analysis), this is in close agreement with the numerical solution to the expression for single elastic scattering used by Armstrong, which yields approximately *Z*^1.35^.

It must be emphasized that exponents are adjusted to obtain the best prediction solely to demonstrate that the variation of backscatter production, in materials of differing composition, can be adequately described by a simple function of atomic number.

## 5. Conclusions

The isotope data presented do not support a mass effect in electron-solid interactions, at least to the fractional percent level. Prediction of electron backscatter in compounds should be based not on the mass fraction, but on the electron fraction, of the constituent elements times the backscatter measured in the respective pure element. Mass-fraction averaging has met some success in predicting electron backscatter because atomic mass happens to vary with *Z* in a manner that partially compensates for nuclear screening of the proton charge in atoms of higher atomic number elements. This screening effect on the proton nuclear charge from the inner orbital electrons, requires an adjustment to the simple electron fraction model.

## Figures and Tables

**Table 1:** Comparison of mass fraction and electron fraction for a number of compounds. The relative difference between the two calculations depends on the *A/Z* ratio of the elements in the compound and is due solely to the effect of the neutron mass of the atom

Compound	Element	Mass fraction	Electron fraction	Relative difference (%)
AuCu	Au	0.756	0.731	−3.3
	Cu	0.244	0.269	10.2
PbS	Pb	0.866	0.837	20.4
	S	0.134	0.163	21.6
NaCl	Na	0.393	0.393	0.0
	Cl	0.607	0.607	0.0
UN	U	0.944	0.929	−1.6
	N	0.056	0.071	26.7
MgO	Mg	0.603	0.600	−0.50
	O	0.397	0.400	0.75
ThSiO4	Th	0.7159	0.6618	−7.6
	Si	0.0867	0.1029	18.6
	O	0.1975	0.2353	19.1
UC2	U	0.983	0.8846	−10.0
	C	0.0917	0.1154	25.8

## Footnotes

1 NIST disclaimer: Certain commercial equipment, instruments, or materials are identified in this paper to foster understanding. Such identification does not imply recommendation or endorsement by the National Institute of Standards and Technology, nor does it imply that the materials or equipment are necessarily the best available for the purpose.
